# Utilization of SiO_2_ Nanoparticles in Developing Superhydrophobic Coatings for Road Construction: A Short Review

**DOI:** 10.3390/molecules30132705

**Published:** 2025-06-23

**Authors:** Nazerke Kydyrbay, Mergen Zhazitov, Muhammad Abdullah, Zhexenbek Toktarbay, Yerbolat Tezekbay, Tolagay Duisebayev, Olzat Toktarbaiuly

**Affiliations:** 1 Renewable Energy Laboratory, National Laboratory Astana (NLA), Nazarbayev University, Kabanbay Batyr 53, Astana 010000, Kazakhstan; nazerke.kydyrbay@nu.edu.kz (N.K.); mergen.zhazitov@nu.edu.kz (M.Z.); muhammad.abdullah@nu.edu.kz (M.A.); yerbolat.tezekbay@nu.edu.kz (Y.T.); tolagay.duisebayev@nu.edu.kz (T.D.); 2 Department of Chemistry, Faculty of Natural Sciences and Geography, Abai Kazakh National Pedagogical University, 13 Dostyk Ave., Almaty 050010, Kazakhstan; zhexenbek.toktarbay@gmail.com

**Keywords:** superhydrophobic coatings, SiO_2_ nanoparticles, road construction, nano-silica application, water-repellent coatings, anti-icing properties, pavement durability, asphalt and concrete coatings, self-cleaning surfaces, UV resistance, mechanical stability of coatings

## Abstract

The application of superhydrophobic (SH) coatings in road construction has attracted growing attention due to their potential to improve surface durability, reduce cracking, and enhance skid resistance. Among various materials, SiO_2_ nanoparticles have emerged as key components in SH coatings by contributing essential surface roughness and hydrophobicity. This review paper analyzes the role of SiO_2_ nanoparticles in enhancing the water-repellent properties of coatings applied to road surfaces, particularly concrete and asphalt. Emphasis is placed on their influence on road longevity, reduced maintenance, and overall performance under adverse weather conditions. Furthermore, this review compares functionalization techniques for SiO_2_ using different hydrophobic modifiers, evaluating their efficiency, cost effectiveness, and scalability for large-scale infrastructure. In addition to highlighting recent advancements, this study discusses persistent challenges—including environmental compatibility, mechanical wear, and long-term durability—that must be addressed for practical implementation. By offering a critical assessment of current approaches and future prospects, this short review aims to guide the development of robust, high-performance SH coatings for sustainable road construction.

## 1. Introduction

The rapid pace of urbanization has significantly increased the demand for durable and sustainable transportation infrastructure. Asphalt and concrete roads form the majority of urban road networks, covering over 30% of surface areas in most cities [1,2,3]. However, pavements are increasingly susceptible to environmental stressors such as moisture, freeze–thaw cycles, and surface icing, especially during winter. These factors not only compromise the structural integrity of roads but also reduce transportation efficiency and increase accident risk due to decreased friction and poorer vehicle control [4]. The traditional solutions to address these issues include passive systems (e.g., snow clearing and de-icing chemicals) and active systems (e.g., heated highways and chemical additives) [5]. Although highly effective, these methods tend to cause surface deterioration, corrosion of reinforcing steel, and increase operational costs [6,7,8,9,10,11,12]. In addition, concrete, which is a prevalent cement-based material, is highly prone to corrosion upon contact with water and aggressive ions, causing premature damage and expensive maintenance actions [13].

In an attempt to improve the durability of road infrastructure and minimize its lifecycle environmental footprint, researchers have suggested a range of solutions, such as enhancing concrete density and using surface treatments or protective coatings to restrict water ingress [14,15,16,17]. Of a number of emerging surface technologies, superhydrophobic coatings (SHCs) have attracted much attention because of their special water-repelling properties that facilitate an easy reduction in moisture ingress and ice formation on the surface. Inspired by natural surfaces such as the lotus leaf, SHCs achieve their function through a synergy between micro-/nanostructured surface topography and the use of low surface energy materials [18,19]. The coatings have demonstrated potential in a wide variety of applications [20,21,22], including self-cleaning [23,24,25,26], anti-icing [27,28,29], corrosion resistance [30,31], anti-biofouling [32,33], drag reduction [34], oil recovery [35], oil/water separation [36], and resilience to harsh environmental conditions [37]. Recent developments in multifunctional coatings, such as tunnel markings enhanced with nano-SiO_2_ and TiO_2_, further highlight the versatility of such materials [38].

With the demand for sustainable and durable construction materials on the rise, SHCs present an encouraging path toward enhanced durability and the sustainability of road infrastructure. For instance, Al-Kheetan et al. illustrated that the use of surface-modified moisture-resistant materials is capable of reducing surface moisture content in mortar and bricks by a considerable amount, thereby enhancing road performance with less degradation [39]. Such observations render surface modification imperative for infrastructure purposes. More and more, scientists are exploring superhydrophobic materials to use as surface coatings for pavements, where their water-repelling properties decrease ice formation, reduce water damage, and extend pavement life [40,41,42,43,44,45,46,47,48]. New studies also report the use of SiO_2_ nanowires in asphalt systems to induce automatic anti-icing properties and influence rheological behavior, supporting a broader use in pavement materials [49].

One of the most widely utilized materials in SHCs is SiO_2_ nanoparticles, which play a significant part in creating the required surface roughness and hydrophobicity [50,51,52]. For example, in addition to traditional silica, thin films of reduced graphene oxide (rGO) obtained through electrostatic spray deposition have also exhibited promising hydrophobic behavior, paving the way for the development of novel materials [53,54]. Silica-based materials are not just versatile and not only can be used as coatings but also can be integrated into novel concrete mixes. This dual purpose allows researchers to assess their effect in reducing water absorption while maintaining compressive strength, for enhancing the durability of concrete structures [55]. Recent nano-SiO_2_@silane/silicate composites have also shown high mechanical stability and impermeability when used in mortar [56].

In road construction applications, SiO_2_ nanoparticles are necessary to create hierarchical roughness for superhydrophobicity, to enhance surface durability, and to provide abrasion resistance [57,58,59]. Their addition also imparts ultraviolet (UV) stability, which is extremely crucial for the materials exposed continuously to sunlight [60,61]. The mechanism of SiO_2_-induced water repellency is based on the enhancement of the water contact angle due to surface texturing, which includes trapping air between the surface and droplet and, thus, reduces water adhesion [62,63,64,65]. This property is highly advantageous for road engineering, as minimizing water infiltration prevents freeze–thaw damage, erosion, and crack propagation [66,67,68,69]. Additionally, SiO_2_ nanocomposites have also been tested in epoxy-based systems to improve the anti-sliding effectiveness of pavements under wet conditions [70].

Apart from this, nano-SiO_2_ surface wetting characteristics have far-reaching applications well outside the construction domain in catalysis, adsorption, polymer reinforcement, optics, bio-imaging, drug delivery, and enhanced oil recovery [71,72,73,74]. This ubiquitous application is testimony to the versatility of the material, its chemical stability, and environmental compatibility. In the case of superhydrophobic coatings, it is even feasible to functionalize SiO_2_ with low-surface-energy materials like fluorosilanes to further lower surface energy. This blend of chemical modification and physical roughness offers the capability to make surfaces that are dry and resistant to degradation by harsh weather conditions [75,76,77,78]. In summary, superhydrophobic coatings, particularly those derived from SiO_2_ nanoparticles, are a novel, green solution to the growing problem of pavement longevity in cities. By enhancing water repellency, mechanical strength, and weather resistance, while reducing the application of energy-consuming or corrosive maintenance methods, these coatings are consistent with modern objectives in green infrastructure development. As the study continues to evolve, such materials can potentially greatly extend the lifespan of roadways while decreasing their environmental footprint.

## 2. Properties of Nano-SiO_2_

SiO_2_ nanoparticles are fine white powders made of high-purity amorphous silica. Their nanoscale size confers a high surface area and strong surface adsorption, thereby ensuring excellent dispersibility in various media. These characteristics are essential in superhydrophobic (SH) coating applications, where uniform nanoparticle distribution ensures consistent surface roughness and water repellency. The low surface energy of properly functionalized SiO_2_ nanoparticles enables the formation of SH surfaces, which are essential for minimizing water adhesion, reducing ice formation, and improving self-cleaning properties in road environments [79,80,81].

Surface energy and dispersibility are particularly important in coating performance. High dispersibility ensures that the nanoparticles are evenly embedded within the binder matrix or surface layer, thereby maximizing the development of hierarchical micro/nano-structured roughness—an essential feature for achieving superhydrophobicity (WCA > 150°). Additionally, low surface energy contributes to the water roll off and ice shedding capabilities, which are vital for maintaining road safety during wet or icy conditions.

**Figure 1:**
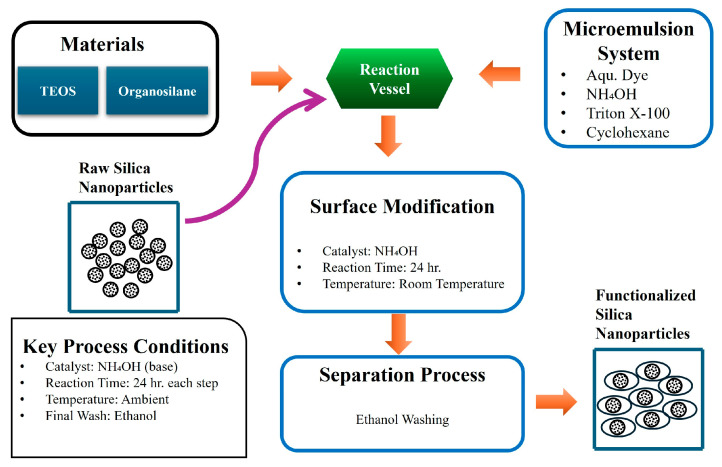
Schematic representation of the surface modification of dye-doped silica nanoparticles using a water-in-oil microemulsion. After 24 h of synthesis, the nanoparticles undergo surface modification via co-hydrolysis of tetraethyl orthosilicate (TEOS) and organosilane reagents for an additional 24 h. Final purification is performed through ethanol washing. Redrawn and adapted from Bagwe et al. [82] for clarity.

Bagwe et al. [82] demonstrated in Figure 1 how surface functionalization using various organosilanes—such as amine, carboxylate, and methyl phosphonate groups—can tailor SiO_2_ properties to reduce aggregation and improve compatibility with different matrices. In road engineering, similar surface treatments enable better bonding with asphalt or concrete substrates, enhancing mechanical integration and durability under traffic and environmental stresses.

In construction materials, nano-SiO_2_ has been shown to enhance mechanical strength and thermal resistance. For example, Ibrahim et al. [83] reported that concrete with nano-SiO_2_ maintained high compressive strength even after exposure to elevated temperatures (400 °C), while Khaloo et al. [84] observed improved flexural and tensile strengths due to reduced microcracks and better particle packing.

Critically, in asphalt applications, SiO_2_-based superhydrophobic coatings have demonstrated excellent long-term performance. Coatings retained water contact angles above 150° after 150,000 wheel abrasions and multiple freeze–thaw cycles, highlighting their resilience in harsh operational conditions [75,78]. These enhancements are directly attributed to the nanoparticles’ optimized surface energy and stable dispersion within the coating matrix, which together contribute to improved skid resistance, reduced water ingress, and extended pavement lifespan.

## 3. Hydrophobic Functionalization of SiO_2_ Nanoparticles

**Figure 2:**
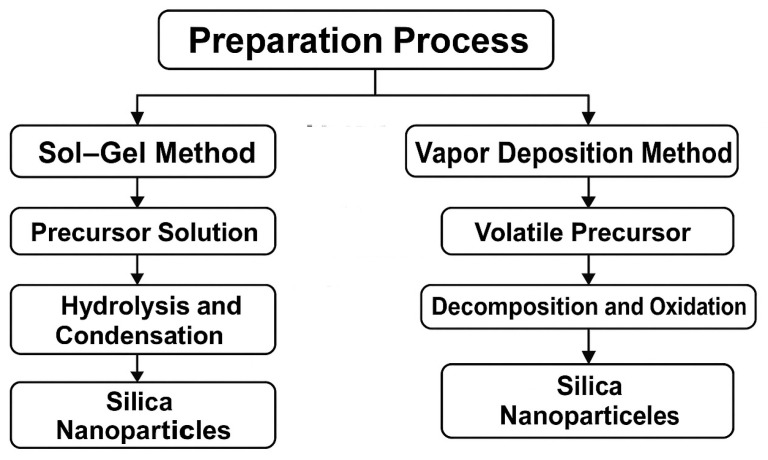
Comparative flowchart showing the preparation of silica nanoparticles via the sol–gel and vapor deposition methods. The sol–gel method involves hydrolysis and condensation of a precursor solution, while the vapor deposition route proceeds through decomposition and oxidation of a volatile precursor.

Silica nanoparticles are frequently modified using alcohols, silanes, amines, fatty acids, and organosilicone compounds. They are typically synthesized and functionalized using various chemical routes. Among these, the sol–gel and vapor deposition methods are the most widely employed for preparing SiO_2_ nanoparticles due to their controllability and scalability. These methods differ in their mechanisms and processing conditions, as illustrated in Figure 2. Table 1 compares effective hydrophobic functionalization methods and application techniques. Among these, silane coupling agents and organosilicone compounds are the most widely used [85,86]. Functionalization not only increases water repellency but also strengthens the coating’s mechanical performance. For instance, silanes like HDTMS and APTES chemically bond with the silica surface, forming a stable low-energy layer that resists water, dirt, and wear. Fuji et al. [87] found that primary alcohols with carbon chains longer than eight significantly enhance nano-SiO_2_ hydrophobicity, while shorter chains require higher grafting rates. This indicates that the chain length and bonding density directly impact coating performance in terms of contact angle and water resistance. Amines such as diethylenetriamine and triethylenetetramine are also used as modifiers [88].

Yang et al. [89] classified superhydrophobic coatings into three types: nanostructured (SCN), microstructured (SCM), and hierarchical (SCH). SCM was created by applying 0.8 kg/m^2^ of liquid, forming micron-sized structures after curing. SCN used 0.14 kg/m^2^ sprayed liquid, producing nanoscale structures through rapid solidification. SCH combined both methods by spraying SCN over pre-formed SCM surfaces.

**Figure 3:**
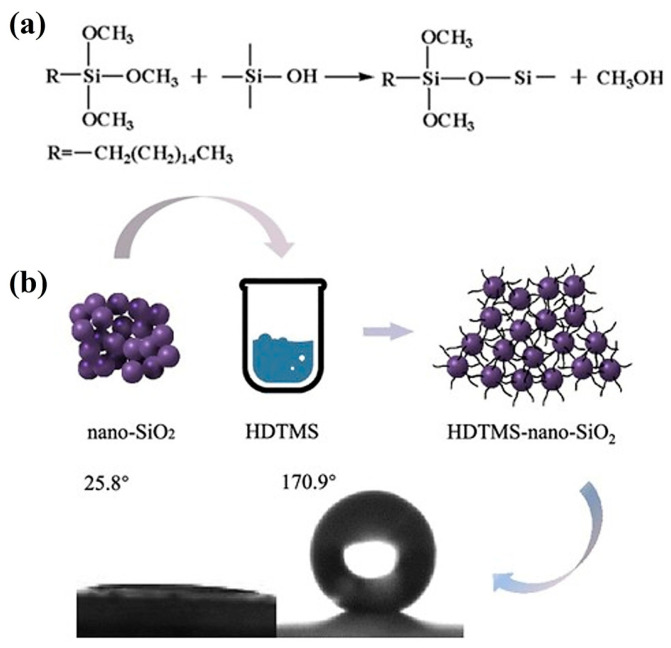
(**a**) Schematic reaction mechanism of hexadecyltrimethoxysilane (HDTMS) hydrolysis and condensation with silica surface –OH groups. (**b**) Surface modification of nano-SiO_2_ with HDTMS resulting in a hydrophobic surface, as indicated by the increase in water contact angle from 25.8° (bare SiO_2_) to 170.9° (HDTMS-functionalized SiO_2_) [91].

Due to environmental concerns with fluorinated silanes, fluorine-free alternatives like APTES and HDTMS are preferred for road use. Ye et al. [90] reported that APTES-based coatings, which avoid environmentally persistent fluorinated compounds, deliver strong hydrophobicity, good mechanical durability, and low toxicity—making them a sustainable choice for road applications. Such properties are essential in real-world road conditions where coatings must withstand rain, traffic loads, and UV exposure. While these alternatives show promise, especially from an environmental standpoint, additional studies on their field performance, aging behavior, and long-term environmental interaction are needed to fully validate their suitability for widespread road construction. Xu B. and Zhang Q. [91] conducted a study in which nano-SiO_2_ was modified using HDTMS in a 0.25:1 ratio. HDTMS-nano-SiO_2_ was prepared by stirring HDTMS and nano-SiO_2_ in ethanol at 90 °C for 1 h, followed by washing, filtration, and drying at 60 °C. The resulting material showed excellent hydrophobicity with a water contact angle of 170.9°. FTIR and 2D-COS confirmed the presence and interaction of -CH_3_, -CH_2_-, and hydroxyl groups. While the FTIR and 2D-COS data confirm chemical bonding, the key practical result is the enhanced water resistance and expected improvement in surface wear tolerance—factors that translate directly into longer service life for treated pavements. The reaction mechanism (a) and the bonding interaction between HDTMS and nano-SiO_2_ are presented in Figure 3.

**Table 1:** Hydrophobic functionalization of SiO_2_ nanoparticles.

	Modification of SiO_2_ NPs	Coating Method	Substrate	WCA	Purpose of the Research Work
1	Polymethyltrifluoropropylsiloxane (Fluorine silicone polymer (PF)) [89]	SCM-dropping SCN-spraying drop + spray	-	160.5°	Comparison of 3 types of substrates (SCM, SCN, and SCH)
2	Ttrimethoxy (1H,1H,2H,2H-heptadecafluorodecyl) silane (fluorinated silane coupling agent (FSCA)) [78]	spraying	-	156°	Prepared fluorinated nano-silica SH coating with anti-icing properties for cement pavement
3	APTES ((3-aminopropyl) triethoxysilan) [90]	spraying for cotton wool –dipcoating method	Glass, steel, filter paper, fabric, wood, and cotton wool	165°	Fluorine-free SH surfaces created using hydrophobic SiO_2_ nanoparticles modified with APTES
4	(3-glycidyloxypropyl) trimethoxysilane (GPTMS), (trihydroxysilyl) propyl methylphosphonate (THPMP), APTES [92]	mixed with bitumen	Mixed with bitumen	-	Modification of SNPs using various silane coupling agents and dual combinations like APTES- THPMP and APTES-GPTMS
5	Dichlorodimethylsilane (DMDCS) [93]	dropping	Copper mesh	155°	The amount of modifier applied plays a crucial role in altering the surface wettability
6	Polydimethylsiloxane (PDMS) [94]	spraying	Glass, paper, and plastic	156.4°	One-step spray coating method using PDMS andSiO_2_ for scalable applications like oil–water separation and self-cleaning
7	Hexadecyltrimethoxysilane (HDTMS) [91]	-	-	170.9°	Modified nano-SiO_2_ achieving a water contact angle exceeding 170°
8	Octadecyltrichlorosilane (OTS) [95]	spraying	Glass slide	165.5°	SiO_2_/silicone rubber nanocomposite coating; icing behavior analysis of water droplets on cold SH surface

Karnati et al. [92] explored the modification of silica nanoparticles (SNPs) using multiple silane coupling agents, including (3-glycidyloxypropyl)trimethoxysilane (GPTMS) and tri(methoxysilyl)propyl methylphosphonate (THPMP), combined with APTES. These dual combinations were mixed with bitumen to develop multifunctional coatings, indicating the feasibility of integrating SH properties into road materials directly. This blending approach improves binder performance and helps extend road durability under thermal and mechanical stress. In contrast, Yan et al. [93] emphasized how the amount of silane modifier—specifically dichlorodimethylsilane (DMDCS)—applied via a dropping method significantly affects the surface wettability of copper mesh substrates, achieving a contact angle of 155°. This highlights the sensitivity of superhydrophobic behavior to precise dosage control during surface treatment. Furthermore, another study [95] introduced a spray-applied nanocomposite coating combining SiO_2_ and silicone rubber, modified with octadecyltrichlorosilane (OTS), which exhibited excellent hydrophobic performance with a contact angle of 165.5°. This coating was evaluated for its icing behavior, particularly in relation to water droplet interactions on cold surfaces, making it highly relevant for anti-icing applications in infrastructure. Such multi-functional coatings provide both water repellency and freeze resistance, reducing maintenance needs and improving winter road conditions. Collectively, these studies underline the critical role of modifier selection, application method, and dosage optimization in achieving durable and functional SiO_2_-based superhydrophobic coatings.

Among the methods summarized in Table 1, PDMS stands out as the most cost effective and scalable hydrophobic functionalization agent for large-scale road applications. This is attributed to its broad availability, relatively low cost compared with other silanes, and simple application via spraying. PDMS-modified coatings demonstrate excellent water repellency, mechanical stability, and long-term durability—maintaining superhydrophobic performance after 72 h of UV exposure, 250 adhesive tape–peel cycles, and immersion in both acidic and alkaline environments [94]. These characteristics are crucial for road conditions, where surfaces are continuously exposed to mechanical stress and weathering. In addition, the coatings retain optical transparency and exhibit strong lipophilicity, supporting their adaptability across various infrastructure uses.

## 4. SiO_2_-Based Superhydrophobic Coatings in Road Construction

Within the scope of SHCs, SiO_2_ nanoparticles are essential components for generating surface roughness and imparting hydrophobicity. Thus, SHC can be developed through a two-component approach, which involves integrating a textured surface structure with a material possessing a low surface free energy to achieve superhydrophobicity. Producing efficient and stable superhydrophobic materials, essential for anti-icing strategies in SHCs, involves combining rough structural features with low-surface free energy (SFE) materials. There are two primary production methods [96,97]. Firstly, low-SFE materials such as polytetrafluoroethylene (PTFE) [44,98] can be applied in nano-structured form [99] onto cement or asphalt surfaces using suitable deposition techniques, such as layer-by-layer (LBL) spraying. Alternatively, surface modifiers can be used to impart low-SFE properties to nanomaterials. Among various options like nano-carbon black [100], carbon nanotubes [101], and nano-silica [48,102], nano-silica stands out as the most suitable for large-scale pavement applications [102].

Table 2 demonstrates SiO_2_-based superhydrophobic coatings utilized in road construction, especially for substrates such as asphalt and concrete. For example, superhydrophobic coatings for cement pavement were developed using fluorinated nano-silica, carbon black, and waterborne epoxy resin. To enhance performance, the study focused on optimizing component composition and spraying dosage through fluorination validation, content analysis, and dosage refinement. Superhydrophobic road coatings have transformative potential for self-cleaning, anti-icing, and corrosion resistance, but their practical use is frustratingly limited by a constellation of interconnected problems. For one, the delicate micro- and nano-structures that give them their extreme water repellency have poor mechanical strength in the face of vehicular traffic’s abrasive onslaught, leading to the rapid degradation of surface functionality [103,104]. Second, the exposure to environmental stressors—UV radiation, wide temperature variations, and chemical stressors such as de-icing salts—can contaminate or chemically break down the coating, once more diminishing performance over time [103,104]. Third, the advanced materials and complicated fabrication techniques involved in these coatings usually result in significantly greater production expenses, and existing manufacturing methods do not easily scale up to the hundreds of square meters demanded by roadway initiatives [103,105]. Last but not least, the absence of widely accepted test parameters for mechanical degradation, environmental exposure, and durability makes it difficult to compare results across studies, as well as to stipulate performance standards needed for certification and regulatory approval [106].

To address these challenges, researchers have proposed several promising approaches. Enhanced fabrication techniques—e.g., the incorporation of fluorosilicone resins within silica nanoparticle matrices—can yield more durable films that are superhydrophobic even after repeated abrasion and chemical exposure [104]. Parallel efforts to develop green precursor materials (e.g., biomass-derived silicas) serve to reduce costs and environmental footprints, thereby improving economic viability and substrate compatibility [105]. As well, the establishment of standardized, quantifiable testing regimes for abrasion resistance, ultraviolet stability, and hydrophobic recovery would facilitate cross-laboratory comparison and encourage technological uptake by providing clear performance benchmarks [106].

Gao et al. designed and evaluated a superhydrophobic coating for cement pavement [78]. The coating’s rough texture was achieved using nano-silica, a widely used engineering nanomaterial. To reduce the surface free energy (SFE), trimethoxy (1H,1H,2H,2H-heptadecafluorodecyl) silane (FSCA) was incorporated, known for its outstanding superhydrophobicity and stability. Waterborne epoxy resin, a non-toxic polymer with excellent processing properties, served as the coating’s base, while carbon black was added to adjust its color. The developed coating demonstrated remarkable engineering performance, improving water droplet repellency by 70%, delaying ice formation by 40%, and enhancing ice layer removal by 40% compared with untreated surfaces.

**Figure 4:**
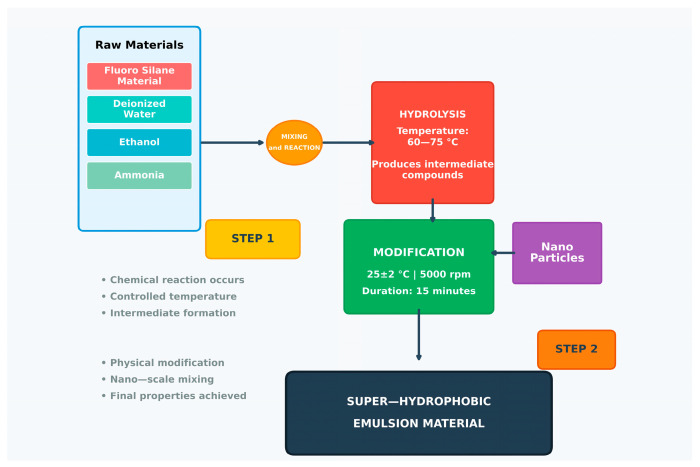
Flow diagram for the synthesis of superhydrophobic emulsion coating material (redrawn based on [78]).

SiO_2_-based superhydrophobic coatings are extensively applied to asphalt surfaces to enhance long-lasting properties such as durability, mechanical stability, self-cleaning, and anti-icing capabilities as shown by Li et al. [75]. In their study SiO_2_-based SH coatings showed strong durability under abrasion and temperature changes. They maintained superhydrophobicity up to 150,000 loading cycles and remained hydrophobic (>110°) after 600,000 cycles. The temperature variations caused minimal changes, with contact angles staying above 150° and sliding angles below 10°, indicating good stability in real-world conditions. In the study, nano-silica particles were fluorinated by dispersing them in an ethanol–water solution, adjusting the pH to 9, adding a fluorosilane agent, and stirring for 24 h before purification. The superhydrophobic coating was then prepared via the sol–gel method by mixing the fluorinated particles with waterborne epoxy resin, black carbon, and a curing agent, followed by spraying onto specimens and curing for six days. The effects of nano-silica and black carbon content, as well as spray dosage, were systematically evaluated to optimize coating performance. Zhao et al. [76] developed an SH coating by modifying nano-SiO_2_ particles with a coupling agent (KH550) and oxysilane. The prepared solution was then sprayed onto asphalt mixture surfaces. Furthermore, applying 40 mL of the SHC—comprising nano-SiO_2_ (30 nm), KH550 (4 mL), MTMS (6 mL), and DMDS (4 mL)—resulted in a peak contact angle of 154.2°. Anti-icing experiments demonstrated that the SHCs effectively prolonged the freezing time of surface water and reduced the adhesion between ice and the pavement. Moreover, Gao et al. [78] outlined a systematic approach for synthesizing a superhydrophobic emulsion coating material, involving two key stages: the hydrolysis process and the modification process as shown in Figure 4. In the hydrolysis stage, a mixture of deionized water, ethanol, and ammonia is prepared, serving as a reaction medium. This mixture undergoes hydrolysis with fluoro silane material at a controlled temperature of 60–75 °C, leading to the formation of hydrolysis products. In the subsequent modification stage, the hydrolyzed products are combined with nano-SiO_2_ particles and processed under specific conditions (25 ± 2 °C, 5000 r/min for 15 min) to enhance their surface properties. This process ensures uniform nanoparticle dispersion and optimal surface functionalization, ultimately yielding a superhydrophobic emulsion material with improved water-repellent characteristics, making it suitable for protective coatings. The superhydrophobic asphalt concrete demonstrated the ability to delay frost formation and progression. The findings showed that the residual ice rate on superhydrophobic asphalt concrete was approximately 21.6% and 48.6% compared with that on conventional asphalt concrete under freezing snow and freezing rain conditions, respectively. Moreover, the SH coatings maintained their durability under friction. After 300 cycles, the contact angle of super-hydrophobic asphalt concrete decreased from 150.7° to 97.4°, but remained >90° even after 1000 cycles. This suggests that the permeable material allows the hydrophobic properties to persist, demonstrating long-term durability and anti-icing effectiveness.

**Figure 5:**
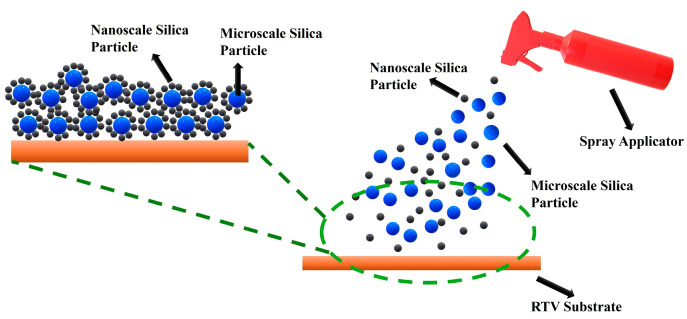
Schematic illustration of micro/nano-structured silica deposition on an RTV surface, inspired by the method described in Peng et al., 2018 (redrawn based on [77]).

The formation of ice on asphalt pavement can pose significant risks to driving safety. To mitigate this hazard, researchers in study [77] developed a superhydrophobic coating for asphalt surfaces. The primary component of the coating is room-temperature vulcanized silicone rubber (RTV). To construct the coating, micro- and nano-sized SiO_2_ particles, modified with a silane coupling agent (KH550), were applied to the RTV surface as shown in Figure 5. SiO_2_ particles were modified using KH550 in an ethanol–water solution (1:10:1 mass ratio), followed by hydrolysis at 40 °C and reaction at 140 °C to form Si–O–Si bonds. The resulting micro/nano-SiO_2_ particles were used to prepare superhydrophobic coatings by spraying them onto an incompletely cured RTV silicone rubber substrate. The RTV was synthesized by blending two components: one containing silicone rubber emulsion, fumed silica, methylsilicone oil, and a catalyst; the other containing ethylsilicate and KH550. Different particle ratios and spray amounts were tested to assess their impact on surface hydrophobicity. Contact angle measurements and surface morphology analysis revealed that the presence of micro- and nano-structured roughness on the coating significantly enhances the water contact angle, improving its superhydrophobic properties. Additionally, to address the issue of oxidation aging in asphalt binders, surface-functionalized silica nanoparticles (SNPs) with (3-aminopropyl) triethoxysilane (APTES) have shown improved dispersion and significantly enhanced anti-aging and rheological properties compared with pristine SNPs [107].

## 5. Conclusions

The incorporation of SiO_2_ nanoparticles into superhydrophobic coatings provides a transformative approach to enhancing the durability and safety of road surfaces. By creating micro- and nano-scale surface roughness, these coatings significantly improve water repellency, reduce water infiltration, and mitigate the adverse effects of freeze–thaw cycles and erosion. They also demonstrate excellent mechanical stability, self-cleaning capability, and anti-icing performance, making them highly suitable for large-scale road applications.

A critical factor in the performance of SH coatings is the hydrophobic modification of SiO_2_ nanoparticles. Numerous studies confirm that silane treatments—especially with HDTMS—achieve superior water contact angles, while PDMS stands out as a cost effective, scalable, and durable modifier for real-world use. General recommendations include modifying nano-SiO_2_ with fluorinated silanes (e.g., FSCA and KH550) to reduce surface free energy and combining them with robust binders like waterborne epoxy or RTV silicone rubber. The optimal dosage levels typically range between 2 and 5 wt% for SiO_2_ in the total coating formulation, with spray applications followed by controlled curing (e.g., 25 ± 2 °C for 6 days) yielding uniform, long-lasting coatings. These formulations have demonstrated a strong resistance to abrasion (e.g., >150,000 loading cycles) and environmental exposure, preserving contact angles above 150° and sliding angles below 10°, even under fluctuating temperatures.

Looking ahead, future research should investigate the long-term field performance of these coatings under various climatic conditions and traffic loads to better understand their durability in real-world settings. Environmental sustainability is another key area; developing eco-friendly, low-VOC, or bio-based modifiers could reduce environmental impact. Additionally, there is a growing need for multi-functional coatings that combine superhydrophobicity with properties such as UV resistance, self-healing, or enhanced abrasion resistance. Optimizing the application methods (e.g., roll-on vs. spray techniques) and evaluating cost–benefit ratios at scale will also be essential to encourage widespread implementation. In summary, SiO_2_-based SH coatings represent a promising advancement in road engineering, with further research poised to unlock their full potential in creating safer, longer-lasting, and more sustainable roadways. While SiO_2_-based superhydrophobic coatings show excellent anti-icing capabilities, their use as passive ice prevention layers differs from conventional de-icing agents. Therefore, large-scale adoption should consider compatibility with regulatory frameworks such as EN 16811-3 to ensure environmental safety, material performance, and compliance in road maintenance applications [108].

## Figures and Tables

**Table 2:** SiO_2_-based SH coatings in road construction.

	**Purpose of the Research Work**	**Preparation Method Utilizing SiO_2_ NPs**	**Coating Method**	**Curing Time on Substrate**	**Substrate**	**WCA**
[75]	De-icing and anti-icing effectiveness	Produced by incorporating nano-SiO_2_ particles at the final stage of the fluoro silane hydrolysis reaction.	Manual or mechanical spraying	-	Asphalt concrete	150.7°
[76]	Anti-icing purposes	SiO_2_ NPs were mixed with silanes (MTMS, DMDS) and coupling agent KH550.	Spraying	Static cultivation for 24 h; dried at 160 °C for 2 h	Asphalt	154.2°
[7]	Robustness of the substrate	Formulation of a hydrophobic solution through the hydrolytic polycondensation of PTES. Synthesis of a nanoparticle solution containing SiO_2_ NPs using the hydrothermal method.	Brushing, spraying	7 days at 21 °C	Concrete-based materials	155°
[77]	Anti-icing and mechanical properties	Functionalization of SiO_2_ NPs using KH550. Application by spraying onto the RTV surface of partially cured silicone rubber.	Spraying	10 min	Asphalt	166.7°
[78]	Anti-icing performance and pavement durability	Fluorination of nano-silica particles with FSCA mixing fluorinated NPs with epoxy resin, black carbon and curing agent to obtain SHS.	Spraying	6 days at room temperature	Cement pavement	156.7°

## Data Availability

No new data were created or analyzed in this study.
